# Impact of teach-back-based training on maternal discharge readiness and the readmission of preterm infants admitted to the NICU: a quasi-experimental study

**DOI:** 10.1186/s12913-025-13926-9

**Published:** 2025-12-26

**Authors:** Mozhgan Mostafanezhad, Fatemeh Valizadeh, Kimia Karami, Rasool Mohammadi

**Affiliations:** 1 Student Research Committee, Nursing and Midwifery School, Lorestan University of Medical Sciences, Khorramabad, Iran; 2 Social Determinants of Health Research Center, School of Nursing and Midwifery, Lorestan University of Medical Sciences, Khorramabad, Iran; 3 School of Nursing and Midwifery, Lorestan University of Medical Sciences, Khorramabad, Iran; 4 Nutritional Health Research Center, Health and Nutritional School, Lorestan University of Medical Sciences, Khorramabad, Iran

**Keywords:** Patient education methods, Discharge planning, Patient readmission, Premature infants, Mothers

## Abstract

**Introduction:**

Mothers of premature infants admitted to the NICU often experience high levels of stress and feelings of unpreparedness for their infants’ discharge. Identifying effective training methods is crucial for enhancing mothers’ ability to provide optimal care at home. This study aimed to determine the effect of teach-back-based training on mothers’ readiness for discharge and the rate of readmission among premature infants admitted to the NICU.

**Methods:**

This quasi-experimental study was conducted on 66 mothers of premature infants hospitalized in the NICU of Shahid Rahimi Hospital, Khorramabad, Iran, during 2022–2023. The control group received standard ward-based training. In the intervention group, nurses applied the teach-back training method after completing a workshop and a two-week pilot implementation. The data collection tools included a discharge readiness checklist and a newborn readmission form. Data were analyzed using the chi-square test and paired and independent t-tests.

**Results:**

Before the intervention, the mean discharge readiness scores did not differ significantly between the groups. However, after the intervention, the mean discharge readiness score in the intervention group (43.12 ± 8.47) was significantly higher than that in the control group (36.60 ± 4.01) (*P* = 0.001). During the one-month follow-up after discharge, the rate of neonatal readmission did not differ significantly between the groups (*P* = 0.054).

**Conclusion:**

Teach-back-based training effectively improved discharge readiness among mothers in NICU. It is recommended that this method be incorporated into care plans and nursing and midwifery curricula. Also, address nursing shortages so that nurses have time for this personalized training.

**Supplementary Information:**

The online version contains supplementary material available at 10.1186/s12913-025-13926-9.

## Introduction

Prematurity, defined as birth before 37 weeks of gestation, is associated with increased morbidity and mortality compared with full-term birth [1, 2]. Globally, between 2010 and 2020, approximately 15% of all preterm births occurred before 32 weeks of gestation [3]. In Iran, where around 5,000 children are born daily, approximately 12% are premature [4]. The specialized care and prolonged hospitalization required for premature infants can impose substantial physical, emotional, and financial burdens on their families [5]. Therefore, preparing families for the discharge and home care of premature infants is a critical component of neonatal care [6].

Discharge readiness refers to the extent to which families feel confident and competent in caring for their infants at home [7]. It reflects the mother’s ability to perform the tasks necessary for the safe and effective care of her newborn following hospital discharge. This concept encompasses knowledge (awareness of care procedures and warning signs), practical skills (e.g., feeding, bathing), confidence in providing care, and the ability to access support resources [8]. Multiple factors influence discharge readiness, including the infant’s medical condition, parental knowledge and skills, the availability of support systems, and the quality of communication and education provided by the healthcare team [9, 10]. Therefore, evaluating and enhancing discharge readiness is an essential component of discharge planning for high-risk infants requiring specialized care after hospitalization [11].

Discharge readiness is a dynamic and multidimensional concept that necessitates continuous assessment and intervention by the healthcare team. The primary goal of discharge planning is to ensure comprehensive preparation and support for the infant and the family during the transition to home care. This approach aims to enhance the quality and safety of care, ultimately improving infant outcomes and family satisfaction [6, 11]. Discharge readiness is associated with important outcomes such as parental satisfaction, infant growth and development, and decrease of readmission rates [12].

Readmission refers to any repeated, unplanned admission of an infant after initial discharge from the NICU and may occur from a few days to several months following discharge [13]. The readmission of premature infants to the neonatal intensive care unit (NICU) is a prevalent, costly, and stressful issue that negatively affects the quality of care and the well-being of both infants and their mothers. Readmission rates for premature infants exceed those of term infants and can adversely influence growth, development, and mother–infant bonding [14, 15]. Inadequate parental preparation for discharge and home care is a leading cause of NICU readmission among premature infants. Therefore, providing effective education and support to parents before and during discharge, emphasizing their active participation in infant care programs, is essential for reducing the risk of readmission [6, 16].

Nurses play a pivotal role in preparing parents for discharge and ensuring continuity of care from the hospital to the home. However, implementing various approaches is warranted to identify optimal methods and practices for empowering parents and enhancing outcomes for preterm infants [17, 18].

In this context, one potential strategy to increase readiness for discharge and reduce the readmission of premature infants in the NICU, as discussed in the literature, involves teach-back-based education. Teach-back is an interactive and effective teaching method in which the nurse, after providing instruction, asks the learner to explain or demonstrate what he or she has learned in their own words. This process helps the educator assess the learner’s level of comprehension and mastery and, if necessary, repeat or simplify explanations to ensure complete understanding. The questions used in this method are open-ended and are intended not to test the learner but to evaluate the quality of the instruction. Teach-back is tailored to the individual’s literacy level and needs and, by increasing the effectiveness of teaching, facilitates self-management and improves health outcomes [19].

This approach emphasizes eliciting requests and input from families, prioritizing their perspectives over traditional provider-driven directives [20]. It also enables healthcare educators to confirm or correct patients’ understanding of issues such as diagnosis, medication, care, and discharge without causing embarrassment. Proper use of this method can help prevent unnecessary and costly readmissions [21].

Empirical evidence supports the notion that continuous teach-back–based training can enhance the comprehension, retention, and application of essential information and skills required by families to care for their infants [20, 22]. Furthermore, teach-back-based training has been shown to increase self-efficacy, defined as the family’s confidence in executing necessary caregiving tasks. Self-efficacy is a critical determinant of discharge readiness, influencing motivation, coping mechanisms, and adherence to care plans among families [23].

However, additional empirical evidence is needed to determine the effectiveness of teach-back-based education in achieving significant objectives such as improving maternal discharge readiness and reducing readmissions of preterm infants. Prior research has predominantly focused on specific aspects of care, such as nutrition or medication management, and has employed varied methodologies and outcome measures. Moreover, most of these studies have been conducted in high-income countries, where the availability and accessibility of healthcare services may differ substantially from those in low- and middle-income settings [10, 12, 20, 24]. Consequently, addressing this research gap is imperative. Therefore, the primary aim of the present study was to assess the impact of teach-back-based training on maternal readiness for discharge and the readmission of premature infants admitted to the neonatal intensive care unit within a resource-limited environment.

## Methods

### Study design

The present quasi-experimental study employed a pretest–posttest design that included both experimental and control groups. The study was conducted during the period of 2022–2023 in the NICU of Shahid Rahimi Hospital in Khorramabad, Lorestan, Iran. This NICU comprises 20 intensive care beds, and mothers are permitted to stay temporarily with their infants to facilitate breastfeeding and bonding. The NICU also includes a designated room where mothers may rest during their hospital stay. Adjacent to the NICU is a 10-bed neonatal unit dedicated to post-NICU care. In this unit, a chair that converts into a bed is provided to allow mothers to remain with their infants 24 h a day. Fathers are permitted to visit for short periods of time; however, they are not allowed extended accompaniment or continuous overnight stays with the infant. Therefore, only mothers were included in the study.

### Sample size and sampling method

The sample size was calculated based on a previous study [25], considering a 95% confidence level (type I error of 5%), a power of 80%, an effect size (d) of 0.72, and a potential attrition rate of 10%. Each group consisted of 33 participants, resulting in a total sample size of 66 mothers.

The inclusion and exclusion criteria were identical for both groups. Inclusion criteria for mothers were: age over 18 years, minimum literacy in reading and writing, proficiency in Persian, a DASS-21 score below 14, and having a premature infant with a gestational age between 28 and 37 weeks and a birth weight of 1000–2500 g.

Mothers were excluded if their infant had a congenital abnormality, was transferred to another center for ongoing treatment, died during the study, or if the mother declined to continue participation. Additional exclusion criteria included mother’s inability to stay at the ward, history of addiction or marital problems, being a single parent, or having previously delivered a premature infant.

To prevent contamination, the two groups were sampled consecutively rather than simultaneously. First, all participants in the control group were selected and enrolled from April to October 2023. Teach-back training for nurses, along with its pilot implementation, took place in August 2023. Sampling for the intervention group then commenced from September to December 2023.

The control group was selected using a convenient sampling method based on the predefined inclusion criteria. After data collection from the control group, variables such as gestational age, sex and birth rank of neonates, and mothers’ education level were examined. To ensure homogeneity, gestational age was categorized into three groups (28–32 weeks, 33–34 weeks, and 35–37 weeks), sex into two categories (female and male), birth rank into two categories (first or second and above), and mothers’ education level into three categories (sub-diploma, diploma, and higher diploma). Following this categorization, sampling for the intervention group was performed according to the frequency distribution of these characteristics in the control group. Participants with matching characteristics were included in the intervention group.

To recruit participants in the study, after hospitalization of each preterm neonate, the mother was examined for inclusion and exclusion criteria by a trained nurse (not a member of the research team). If she met the inclusion criteria, after a relatively stable condition of the neonate, she was informed about the study and invited to enter the study with emphasizing confidentiality and other rights. If she was willing to participate, informed written consent was obtained, and questionnaires were completed.

Nurses participating in the study were required to meet the following criteria: willingness to engage in the study, at least a bachelor’s degree in nursing, and a minimum of three months of work experience in the neonatal unit. Nurse selection followed predefined criteria, and efforts were made in collaboration with the department head and educational supervisor to minimize staff transfers within the unit during the study period.

### Data collection tool

#### Demographic information form

The demographic information form included sections on mothers, premature infants, and nurses. For mothers, demographic variables comprised age, education level, occupation, family income, history of illness, history of abortion, type of delivery, husband’s occupation and education level, and the presence of a familial supporter for helping mother in infant care. Infant-related demographic characteristics included gestational age, birth weight, sex, duration of hospital stay, birth rank, and Apgar score. For nurses, the investigated characteristics included age, marital and employment status, education level, and parental status. The form was thoroughly reviewed, revised, and approved by faculty members of the Khorramabad Nursing and Midwifery Faculty. Mothers and nurses completed these forms under the supervision of a trained nurse (not a member of the research team) prior to the intervention.

#### Discharge readiness checklist

The discharge readiness checklist, developed by Dashti et al., consists of 28 items divided into two subscales. The first 12 items assess the infant’s physical condition and general health, while the remaining 16 items focus on the mothers’ knowledge and skills related to newborn care. Items are scored on a 3-point Likert scale: No = 1, Unfavorable = 2, and Favorable = 3. The total score ranges from 28 to 84, with higher scores indicating a higher level of discharge readiness. Face validity was confirmed by 15 faculty members, including six from the Faculty of Management and Information and nine from Shahid Beheshti Faculty of Midwifery and Nursing. The content validity index (CVI) was 0.99, and reliability, assessed by the intraclass correlation coefficient (ICC), was 0.88 [26]. The checklist was completed in person by a trained nurse (not a member of the research team) through structured interviews and observation of maternal performance. The first assessment was conducted within the first 1–2 days of hospitalization, and the second assessment was performed 1–2 days before discharge.

#### Readmission and outpatient visits form

This form recorded information regarding the rate and reasons for neonatal readmissions and unplanned outpatient visits to clinics or to physicians’ office within one month after the discharge. Data were collected weekly via telephone calls to the mothers by a trained nurse (not a member of the research team) for four consecutive weeks.

#### DASS-21 scale

The DASS-21 is a validated psychometric instrument comprising 21 items that assess depression, anxiety, and stress over the past week. Each subscale contains seven items, with responses recorded on a 4-point scale. Its validity and reliability have been confirmed in the Iranian population [27]. In the present study, the DASS-21 was used solely for initial screening and as an inclusion criterion; participants with a total score below 14 were eligible for inclusion. Scores above this threshold indicated severe depression, anxiety, or stress, and those individuals were not included in the study.

### Procedures

The study was conducted in five distinct stages.

#### Control group sampling

To prevent information transfer between groups, sampling for the control group was conducted first. Participants in the control group received standard education provided by ward personnel through routine methods. During this phase, a designated training officer delivered routine education to mothers at the time of discharge. The training covered breastfeeding techniques, expressing breast milk, feeding with breast milk or specialized formula, maternal nutrition, mother–infant bonding through nurturing care, infant massage, personal hygiene, and proper milk storage and feeding practices.

Education was provided orally, in group sessions, and supplemented with written materials in the form of an instructional leaflet, which was given to the mother or accompanying caregiver at discharge. The leaflet included guidance on the infant’s diet and highlighted warning signs such as jaundice, lethargy, fever, cyanosis, and vomiting, instructing caregivers to seek medical attention if these symptoms occurred. The training officer also emphasized the importance of follow-up screenings, including a second thyroid test, audiometric examination within the first 24–48 h post-discharge, and optometric assessment via referral to a health center. When additional evaluations—such as brain scans or magnetic resonance imaging, bilateral hip ultrasound, or retinopathy of prematurity screening within the first 28 days—were necessary, families were provided with specific instructions.

Caregivers, primarily mothers, were educated on recognizing inadequate weight gain in their infants and advised to readmit the infant if required. Post-discharge follow-up, including breastfeeding support, monitoring for complications related to the infant’s special care admission, and assessment of parental caregiving, was conducted by the designated education personnel within the first 24–48 h after discharge. Mothers were encouraged to contact the ward with any questions or concerns.

#### Preparation of educational package

The educational package was developed based on comprehensive research and an extensive review of the literature. A wide range of resources was utilized in preparing this package, including national (Persian) guidelines and service packages on newborn care, breastfeeding, kangaroo mother care, vaccination, and other relevant sources [4, 6, 7, 24, 25, 28–34].

The content of the educational package focused on training nurses about the importance of preparing mothers for the discharge of premature infants, reducing NICU readmission rates, and enhancing mothers’ knowledge and skills to ensure readiness for infant care at home. The overarching aim was to empower mothers to actively engage in the care process and achieve readiness for NICU discharge.

In addition to routine NICU training, the package provided detailed guidance on essential care practices for premature infants, including hand hygiene, proper diaper changing techniques, milk preparation and storage, pacifier or finger-sucking exercises, breastfeeding techniques, burping procedures, feeding frequency and assessment of feeding adequacy, facilitating infant sleep and safe sleeping positions, soothing methods, kangaroo mother care, infant massage, bathing, post-discharge care, resuscitation according to prematurity or medical conditions, social support for exclusive breastfeeding, skin care, maintaining a healthy maternal diet, promoting healthy sleep, recognizing potential danger signs, administering medications, monitoring normal growth and development, and vaccination.

The content validity of the educational package was rigorously reviewed and validated by seven experts, including five faculty members specializing in maternal and newborn health nursing education, the NICU head nurse, and a senior NICU nurse. These experts evaluated the relevance and necessity of each section of the training content. All suggested modifications were incorporated, and the review process for content validity was repeated multiple times, resulting in the final validated educational package [Content Validity Ratio (CVR] = 0.99; Content Validity Index (CVI) = 1).

#### Teaching nurses the teach-back-based training method

All nurses in the department participated in a comprehensive training program covering the content of the educational package. The training was delivered through two virtual sessions followed by two face-to-face sessions, during which nurses were instructed on how to effectively deliver the educational content to mothers using the teach-back method. During the training, the principal investigator initially demonstrated the teach-back method to the nurses. Nurses were then prompted to articulate the training content in their own words. Any ambiguities or misunderstandings were addressed and clarified by the researcher. This iterative process continued until nurse demonstrated consistent and comprehensive mastery of the material, ensuring the effectiveness of their training.

The training was provided by the principal investigator, a pediatric nursing master’s student with rs of clinical experience in maternal and neonatal care. She had previously completed a comprehensive teach-back training workshop and received certification, equipping her with the theoretical knowledge and practical skills necessary to design and implement teach-back–based education. Supervising researchers, including the study supervisor and consultant professors with specialized expertise in maternal and neonatal nursing education, oversaw all stages of the research, ensuring scientific rigor and quality through expert guidance and feedback.

#### Pilot implementation of teach-back-based training by nurses

In the fourth stage, following completion of the training program, nurses applied the teach-back–based method to instruct mothers over a two-week pilot period. During this period, the researcher was present in the NICU conducted at least two evaluations for each nurse to assess the accurate application of the teach-back method. To this end, adherence to ten key elements of this method, including maintaining a compassionate tone and supportive attitude, establishing eye contact and comfortable body language, using simple and understandable language, encouraging the mother to respond in her own words, asking open-ended questions without creating a sense of embarrassment, avoiding closed questions, emphasizing the responsibility of providing clear explanations, repeating and re-evaluating in case of incorrect understanding, using written materials appropriate to the mother’s needs, and documenting the education provided [35], were evaluated. Each nurse’s performance was carefully evaluated against these criteria, and any necessary corrections or additional guidance were provided to ensure adherence and proficiency in the teach-back method.

#### Implementation of teach-back-based training for the intervention group

In the intervention group, nurses first provided mothers with the written educational package to read and scheduled at least four individualized 30-minute educational sessions to teach its content. These sessions were conducted in person using the teach-back method, beginning in the initial days of the infant’s hospitalization, in the designated room for mothers’ presence and rest in the NICU and the adjacent post- NICU neonatal ward.

The nurse delivered the educational content in simple, easily understandable language, avoiding medical jargon. Following the instruction, the mother was asked to retell the information in her own words, allowing the nurse to assess her comprehension and learning. Any ambiguities or misunderstandings were clarified, and the education continued until the mother demonstrated complete understanding. To enhance practical learning, certain skills—such as administering medication using a syringe, dropper, or spoon—were demonstrated hands-on. Other procedures, such as infant bathing, were explained using educational videos to facilitate understanding and skill acquisition.

### Data analysis

Descriptive statistics, including absolute and relative frequency distributions, measures of central tendency, and measures of dispersion, were used to summarize and categorize the data. To address the primary research objectives, inferential statistical tests—including the chi-square test, Fisher’s exact test, independent t-test, and paired t-test—were employed to compare between-group and within-group differences before and after the intervention. The significance level for all statistical tests was set at *P* < 0.05.

## Results

Between-group comparisons of sociodemographic and clinical characteristics of mothers and premature infants at baseline revealed no significant differences, indicating that the groups were homogeneous (*P* < 0.05) (Table 1). Additional demographic characteristics are provided in Appendix 1.

**Table 1:** Demographic characteristics of mothers and neonates at baseline (*N* = 66)

Characteristics	Control (*n* = 33) Mean ± SD or N (%)	Intervention (*n* = 33) Mean ± SD or N (%)	*P* value
Neonates’ gestational age at birth (weeks)	33.91 ± 2.21	33.66 ± 2.14	0.653*
Neonates’ weight at discharge (grams)	1931.21 ± 390.21	1988.94 ± 436.28	0.448*
Neonates’ age at discharge (days)	17.72 ± 8.28	14.73 ± 7.57	0.573*
Mothers’ age (years)	30.94 ± 6.48	31.64 ± 5.91	0.650*
Mothers’ education level			
High school	13 (39.5%)	10 (29.3%)	0.511**
Diploma	16 (48.5%)	16 (48.5%)	
Above diploma	4 (12.1%)	7 (21.2%)	
Number of children			
One	13 (39.4%)	14 (42.5%)	0.598**
Two	7 (21.2%)	8 (24.2%)	
Three or more	12 (36.4%)	11 (33.3%)	

*Independent t-test **chi-square test

At baseline, no significant between-group differences were observed in the mean discharge readiness scores or their subscales among mothers of premature infants hospitalized in the neonatal intensive care unit (*P* > 0.05). However, following the intervention, a significant between-group difference emerged (*P* < 0.05). In the intervention group, a significant within-group increase was observed in the mean scores of mothers’ discharge readiness and all subscales from baseline to post-intervention (*P* < 0.001). In the control group, a significant within-group increase was also noted in the overall discharge readiness scores (*P* < 0.001) and in mothers’ educational ability (*P* < 0.001); however, the increase in the infants’ general health subscale was not statistically significant (*P* = 0.184) (Table 2).

**Table 2:** Between-group and within-group comparison of mothers’ discharge reediness mean at baseline and after the intervention (*N* = 66)

variables		Intervention Mean ± SD	Control Mean ± SD	*P* value*
Discharge readiness	Before the intervention	32.27 ± 2.05	32.93 ± 2.59	0.251
	After the intervention	43.12 ± 8.47	36.60 ± 4.01	< 0.001
	P value**	< 0.001	< 0.001	
Infant’s general health	Before the intervention	14.78 ± 1.24	14.91 ± 1.07	0.673
	After the intervention	18.75 ± 3.78	14.81 ± 1.08	< 0.001
	P value**	< 0.001	0.184	
Mother’s Knowledge& skills of infant care	Before the intervention	17.48 ± 1.32	18.03 ± 2.20	0.228
	After the intervention	24.36 ± 5.85	21.78 ± 3.86	0.039
	P value**	< 0.001	< 0.001	

*Independent t test ** Paired t test

In the first week after discharge, the readmission rate was 3.0% in both the control and intervention groups, with the control group case attributed to diaphragmatic hernia and the intervention group case attributed to pneumonia. No statistically significant difference was observed between the groups (*P* > 0.05). Subsequent follow-ups showed no additional hospitalizations in either group up to one-month post-discharge. Similarly, the rates of outpatient visits to physicians’ offices and clinics did not differ significantly between the groups (*P* > 0.05) (Table 3).

**Table 3:** Between-group comparison of outpatient visits to doctor’s office and clinic

Time	Intervention *N* (%)	Control *N* (%)	*P value
First week	0 (0.0%)	1 (3.0%)	0.5
Second week	2 (6.1%)	3 (9.1%)	0.693
Third week	1 (3.0%)	3 (9.1%)	0.307
Fourth week	0 (0%)	2 (6.1%)	0.246
From baseline to first month’s end	3 (9.1%)	9 (27.3%)	0.054

*Fisher’s exact test

In the control group, outpatient visits during the first week included one because of presence blood in the stool. In the second week, one due to feeding difficulties and another for an eye problem. During the third week, two patients sought medical attention for constipation and bloating, and one patient reported urinary difficulties. In the fourth week, two patients visited the because of constipation and flatulence.

In the intervention group, outpatient visits were recorded in the second and third weeks. During the second week, one patient was visited for breathing problems, and in the third week, one patient was treated for jaundice, while another sought medical attention for constipation and abdominal bloating. Notably, no outpatient visits were reported in the first or fourth week in the intervention group.

## Discussion

The findings indicated that, prior to the intervention, there were no significant differences between the intervention and control groups in terms of mothers’ overall discharge readiness or its subscales for premature infants in the neonatal intensive care unit. However, following the intervention, a notable between-group difference emerged, with the intervention group demonstrating significantly higher mean discharge readiness compared to the control group. In contrast, no statistically significant difference was observed between the groups regarding infant readmissions post-intervention. Notably, the intervention group exhibited a clinically meaningful reduction in the number of outpatient visits for infants compared with the control group. Overall, these results suggest that teach-back-based training positively influences discharge readiness and associated outcomes.

These findings align with previous research in the domain of family-centered care and parental education for premature infants. For example, Shahraki et al. demonstrated that family-centered educational and supportive interventions enhance parents’ sense of competence and their ability to fulfill a caregiving role [36]. Potential mechanisms underlying these effects include providing structured, practical education, positioning mothers at the center of care, addressing their informational and emotional needs, and offering opportunities for supervised practical practice. The present study reinforces that when education is individualized, structured, and delivered in a clinical setting, the transfer of learning and mothers’ confidence in caring for their premature infant post-discharge are enhanced.

The outcomes of this study support the hypothesis that teach-back-based training contributes to the improvement of discharge readiness among mothers of preterm infants in the NICU. Baseline comparisons indicated no significant differences between groups in maternal discharge readiness or infant readmissions, highlighting the comparability of groups prior to the intervention and underscoring the role of the teach-back intervention as a key factor influencing the observed outcomes.

Several studies support the findings of the present study. In this context, Hariati et al. reported that the implementation of teach-back-based health education and supportive systems enables nurses to empower mothers in caring for premature infants, thereby enhancing discharge readiness and promoting maternal independence in infant care. The authors emphasized the importance of a multidisciplinary team, clear hospital policies, and strengthened nursing competence to ensure a successful transition from the NICU to home, highlighting the critical role of parental education and structured discharge processes [29].

Similarly, Amini et al. demonstrated that teach-back-based education improves the health literacy of mothers with premature infants and enhances maternal self-efficacy in childcare and disease prevention. These outcomes play a pivotal role in preparing mothers for discharge and reducing hospital readmissions [30]. Cheng et al. further indicated that the teach-back method positively influences postpartum health behaviors, leading to improved maternal and infant health outcomes [20].

Sohrabi’s study advocated the implementation of structured educational programs and the provision of educational packages by nurses as effective strategies to enhance maternal adaptation to the motherhood role and improve newborn care [37]. Conversely, Sherfi et al. reported no significant differences between intervention groups using mobile application–based and face-to-face education approaches post-intervention in terms of infant weight gain and mother–baby attachment subscales. Nevertheless, both groups exhibited significant improvements in infant weight compared with pre-intervention levels [38].

No statistically significant differences were observed in infant readmission rates between the intervention and control groups. These findings are consistent with previous studies by Kandula et al., Assareh et al., and Roohani et al., which reported that educational interventions did not significantly reduce the number of re-hospitalizations among cardiac patients [39–41]. Similarly, a randomized clinical trial conducted by Karbandi et al. indicated that implementing a mother’s empowerment program had no significant effect on the readmission rate of premature infants [31].

In contrast Alaee Karahroudy study demonstrated that implementing a parent empowerment program effectively reduced both the re-hospitalization rate and the length of hospital stay for premature infants in the NICU [32]. Additionally, Shermont et al. reported an 8% reduction in seven-day readmissions and a 10% reduction in 30-day readmissions among children following teach-back-based training [42]. Krupp et al. applied the teach-back method to educate families of children with asthma, resulting in decreased readmission rates over a 12-month follow-up period [43]. Furthermore, a systematic review by Oh et al., investigating the effectiveness of teach-back-based discharge training on 30-day readmissions, reported a substantial 45% reduction in 30-day readmission rates [44]. Variations in outcomes across studies may be attributed to differences in demographic factors such as parental age, number of children, occupation, income level, and variations in the duration of follow-up after discharge.

However, teach-back-based training substantially reduced the number of outpatient visits to physicians’ offices and clinics by approximately one-third compared with the control group. This finding can be explained by the challenges and uncertainties mothers of premature infants face upon discharge, as they assume responsibility for their infants’ care at home. Effective interventions are therefore essential to prepare mothers for this transition, addressing both their educational and emotional needs. Teach-back-based training is one such intervention, employing a communication method in which the learner repeats or demonstrates the acquired information in their own words. This approach allows healthcare providers to assess understanding and clarify any misconceptions [45].

Several studies have demonstrated the positive impact of teach-back-based education on maternal discharge readiness and on reducing readmissions among premature infants in the NICU. Teach-back-based education enhances mothers’ self-confidence and satisfaction with the care and instruction provided by healthcare professionals, thereby increasing adherence to discharge plans and follow-up care [33, 46]. Additionally, it improves mothers’ knowledge and skills in home care for premature infants, covering essential aspects such as breastfeeding, infection prevention, jaundice management, and temperature regulation. This improvement in care quality consequently reduces the need for additional health services [20].

Moreover, teach-back-based training decreases the risk of complications and adverse outcomes in premature infants, including dehydration, hypothermia, sepsis, and hyperbilirubinemia, thereby promoting infant health and survival while simultaneously reducing healthcare costs [29]. It also contributes to lower rates of readmissions and emergency visits for premature infants, which are common and resource-intensive. This reduction not only alleviates pressure on the healthcare system but also decreases stress for families [34, 47].

The findings of this study have important implications for the care of premature infants and their mothers. Teach-back-based education has emerged as an effective intervention to improve maternal outcomes, enhance readiness for discharge, and increase satisfaction within this vulnerable population. The implementation of teach-back-based training has the potential to reduce healthcare costs and mitigate the burden associated with the readmission of preterm infants. Therefore, incorporating teach-back-based education into routine practice and evaluating its effectiveness across diverse clinical settings, tailored to the specific needs and preferences of mothers and their infants, is strongly recommended.

The primary limitation of this study lies in its quasi-experimental design, which did not involve randomization of participants between the two groups. Additionally, the follow-up period was limited to one month. Future research is encouraged to employ randomized controlled trial designs with extended follow-up durations to provide a more comprehensive understanding of the long-term effects of teach-back-based interventions.

## Conclusion

Teach-back-based training effectively increased discharge preparation for mothers of preterm infants in the NICU and significantly reduced outpatient visits. It is recommended that nurses incorporate this approach into NICU care, that nursing and midwifery curricula be revised, and that administrators address nurse shortages to provide sufficient time for person-centered training. These measures will improve discharge quality and provide better outcomes for mothers, infants, and health facilities.

## Supplementary Information

Below is the link to the electronic supplementary material.

Supplementary Material 1

## Data Availability

The datasets generated and analyzed during the current study are available from the corresponding author at reasonable request.

## Abbreviations

NICU: Neonatal Intensive Care Unit
CVI: Content Validity Index
ICC: Interclass Correlation Coefficient
NVD: Normal Vaginal Delivery
MRI: Magnetic Resonance Imaging
DASS-21: The Depression, Anxiety and Stress Scale – 21
SD: Standard Deviation
N: Number
