# Supplementary material for: Impact of teach-back-based training on maternal discharge readiness and the readmission of preterm infants admitted to the NICU: a quasi-experimental study
Source: BMC Health Serv Res. 2025 Dec 26;26:156. doi: 10.1186/s12913-025-13926-9 (PMC12849624; doi:10.1186/s12913-025-13926-9)
Supplement: Supplementary file 1 — Supplementary Material 1 [file 12913_2025_13926_MOESM1_ESM.docx]

| ***Table 1.*** *Neonates' sociodemographic and clinical characteristics at baseline (N = 66).* | | | | |
| --- | --- | --- | --- | --- |
| P value | Groups | | *Characteristics* | |
|  | Control  Mean ± SD/ N (%) | Experimental  Mean ± SD/ N (%) |  |  |
| *0.653 | 33.91 ± 2.21 | 33.66 ± 2.14 | Fetal age at birth (weeks) | |
| *0.906 | 7.82 ± 0.91 | 8.39 ± 0.79 | Apgar score | |
| *0.546 | 16.91 ± 7.88 | 15.67 ± 8.72 | Hospitalization duration (days) | |
| *0.448 | 1931.21 ± 390.21 | 1988.94 ± 436.28 | Discharge weight (grams) | |
| *0.573 | 17.72 ± 8.28 | 14.73 ± 7.57 | Age at discharge (days) | |
| **0.805 | 14 (42.4%) | 16 (48.5%) | Girl | Gender |
|  | 19 (57.6%) | 17 (51.5%) | Boy |  |
| **0.942 | 16 (48.5%) | 13 (39.4%) | First | Birth rank |
|  | 17 (51.5%) | 20 (60.6%) | Second and above |  |

*independent t test ** Fisher's exact test

| ***Table 2.*** *Mothers' sociodemographic and clinical characteristics at baseline (N = 66).* | | | | |
| --- | --- | --- | --- | --- |
| P value | Groups | | Groups | |
|  | Control  N (%) | Experimental  N (%) |  |  |
| *0.511 | 13 (39.5%) | 10(29.3%) | High school | **Mother's education** |
|  | 16(48.5%) | 16(48.5%) | Diploma |  |
|  | 4 (12.1%) | 7 (21.2%) | Above diploma |  |
| *0.427 | 28 (84.8%) | 31 (93.9%) | housewife | **Mother's job** |
|  | 5 (15.2%) | 2 (6.1%) | employed |  |
| *0.150 | 28 (84.8%) | 22 (66.7%) | Diploma | **Husband's education** |
|  | 5 (15.2%) | 11 (33.3%) | Above diploma |  |
| *0.792 | 5 (15.2%) | 4 (12.1%) | Employed | **Husband's job** |
|  | 23 (69.7%) | 22 (66.7%) | Freelance |  |
|  | 5 (15.2%) | 7 (21.2%) | Other |  |
| *0.347 | 13 (39.4%) | 14 (42.5%) | One | **Number of children** |
|  | 13 (39.4%) | 8 (24.2%) | Two |  |
|  | 7 (21.2%) | 11 (33.3%) | Three and more |  |
| **0.598 | 12 (36.4%) | 9 (27.3%) | Yes | **History of abortion** |
|  | 21 (63.6%) | 24 (72.7%) | No |  |
| **0.999 | 6 (18.2%) | 5 (15.2%) | NVD | **Type of delivery** |
|  | 27 (81.8%) | 28 (84.8%) | Cesarean section |  |
| *0.249 | 13 (39.4%) | 16 (48.5%) | First time | **Number of births** |
|  | 12 (36.4%) | 6 (18.2%) | Second time |  |
|  | 8 (24.2%) | 11 (33.3%) | Third time and more |  |
| **0.339 | 4 (12.1%) | 8 (24.2%) | Yes | **History of illness** |
|  | 29 (78.9%) | 25 (75.8%) | No |  |
| **0.443 | 23 (69.7%) | 19 (57.6%) | Yes | **Presence a supportive person in family** |
|  | 10 (30.3%) | 14 (42.4%) | No |  |
| ***0.650 | 30.94 ± 6.48 | 31.64 ± 5.91 | **Age (**Mean ± SD) | |

*Chi-square test **Fisher's exact test ***Independent t test
